# Mechanisms and therapeutic strategies to reveal and overcome T-cell dysfunction in gastric cancer: translation from basic research to clinical application

**DOI:** 10.3389/fimmu.2025.1681539

**Published:** 2025-09-30

**Authors:** Huanyu Luo, Jianxi Wu, Yalan Yan, Danqi Xu, Jieying Zhang, Xuancheng Zhou, Guanhu Yang, Xiaolin Zhong

**Affiliations:** ^1^ Department of Gastroenterology, Affiliated Hospital, Southwest Medical University, Luzhou, China; ^2^ Southwest Medical University, Luzhou, China; ^3^ First Teaching Hospital of Tianjin University of Traditional Chinese Medicine Tianjin, Tianjin, China; ^4^ Department of Specialty Medicine, Ohio University, Athens, OH, United States

**Keywords:** T-cell exhaustion, immune checkpoint, biomarkers, cancer immune evasion, gastric cancer, immune microenvironment

## Abstract

T cells play a central role in the immune response to gastric cancer, and their dysfunction directly contributes to immune escape from the tumor and limits the efficacy of immunotherapy. The immune microenvironment of gastric cancer consists of a wide range of cells and molecules, and this complex and dynamic environment exerts profound inhibitory effects on T cell function. upregulation of PD-1, CTLA-4, and other inhibitory molecules is a key mechanism of T cell depletion, and metabolic reprogramming and chronic antigenic stimulation further weaken the anti-tumor activity of T cells. In recent years, PD-1/PD-L1 inhibitors have demonstrated some efficacy in gastric cancer, but the problem of drug resistance remains prominent. To address these challenges, combinatorial therapeutic strategies have gradually become the focus of research, especially combining immune checkpoint inhibitors with chemotherapy, radiotherapy, or targeted therapy to enhance the antitumor effect of immunotherapy. This review delves into the molecular mechanisms of T-cell depletion and its impact in gastric cancer immunotherapy, and analyzes the potential application of biomarkers in predicting treatment response. By comprehensively analyzing T-cell depletion and the immune microenvironment in gastric cancer, this paper provides a theoretical basis for the development of future personalized combinatorial therapeutic strategies, with the aim of improving patient prognosis and enhancing the overall therapeutic efficacy.

## 1 Introduction

Gastric cancer is the fifth most common malignant cancer worldwide and the fourth leading cause of cancer-related deaths (1). According to the World Health Organization (WHO), there will be approximately 1 million new cases of gastric cancer worldwide in 2020, accounting for 5.6% of all cancer cases, the incidence of gastric cancer in East Asia (China, Japan, Korea, etc.) is much higher than that in Europe and the United States, with significant geographical differences (2, 3). In addition to this, the incidence of gastric cancer also has obvious gender distribution differences, and the incidence of gastric cancer in men is approximately twice as high as that in women (1, 4). Adenocarcinoma is the most common histologic subtype of gastric cancer, in addition to mucinous adenocarcinoma, indolent cell carcinoma, and undifferentiated carcinoma (5). Gastric cancer has a rapid progression and is prone to lymph node metastasis and distant metastasis, resulting in a poor prognosis (6). Therefore, the study of the pathogenesis of gastric cancer and its therapeutic strategies is essential to improve clinical prognosis.

T cells are one of the core components of the adaptive immune system that recognize and kill tumors (7).CD8+ cytotoxic T lymphocytes (CTLs) directly kill tumor cells by recognizing tumor antigens and releasing perforin and granzyme (8, 9). And CD4+ helper T cells coordinate the immune response by secreting cytokines (10). The functional soundness of T cells is an important guarantee to maintain anti-tumor immune surveillance (11). However, in gastric cancer and other solid tumors, T cells often exhibit dysfunction (e.g., depletion or heterogeneity), and their antitumor efficacy is greatly diminished (12). A distinctive feature of T-cell dysfunction is the upregulation of immune checkpoint molecules, such as programmed death receptor-1 (PD-1) and cytotoxic T-lymphocyte antigen-4 (CTLA-4), the expression of which leads to T-cell suppression which in turn promotes tumor evasion of immune surveillance (13–15). In addition, chronic antigenic stimulation, metabolic abnormalities, and suppressive cytokines in the tumor microenvironment contribute to the gradual loss of effector function of T cells, further exacerbating immune escape from the tumor (16). Therefore, an in-depth understanding of the dysfunctional mechanisms of T cells in gastric cancer immunity can help develop novel immunotherapeutic strategies (17, 18).

This article delves into the molecular mechanisms underlying T cell dysfunction in gastric cancer and explores potential therapeutic strategies. By integrating basic and clinical research findings, we elucidate the intricate role of T cells in gastric cancer immunity. We focus on T cell depletion and heterogeneity, and highlight the potential application of biomarkers in predicting therapeutic response. Ultimately, we aim to provide novel insights for the development of future personalized immunotherapy strategies.T-cell dysfunction in gastric cancer.

### 1.1 Mechanisms of T-cell exhaustion

T-cell exhaustion is a state in which T cells gradually lose their effector function under continuous antigenic stimulation (19). Depleted T cells exhibit decreased proliferative capacity, decreased secretion of effector molecules (e.g., IFN-γ, TNF-α, IL-2), and high expression of multiple inhibitory receptors (PD-1, CTLA-4, TIM-3, etc.) (20). In gastric cancer, T cell depletion is one of the important mechanisms of tumor immune escape (21).

#### 1.1.1 Immune checkpoint inhibitory pathways

The PD-1/PD-L1 pathway is one of the most well-studied mechanisms of T-cell exhaustion (22). Programmed Cell Death Protein 1 (PD-1), an inhibitory receptor on the surface of T-cells, is usually highly expressed in response to chronic antigenic exposure and inhibits T-cell activity by binding to its ligand, PD-L1, thereby preventing its killing of tumor cells (23). Gastric cancer cells often exploit this pathway to evade immune surveillance by overexpressing PD-L1 (24). This inhibitory mechanism not only plays a role in depleting T cells, but is also upregulated during acute T cell activation to prevent T cell overreaction. In acute infection models, inhibitory receptors including PD-1 and Cytotoxic T-lymphocyte-associated protein 4 (CTLA-4) are up-regulated to counteract the activating effects of T-cell receptor (TCR) and co-stimulatory signals to maintain immune homeostasis (25).

During immune depletion, the presence of PD-1 was not essential. However, its existence notably diminished the extent of depletion and to some extent, prevented cells from becoming overstimulated. This implies that even in the context of depleted T cells, PD-1 may still be exerting a protective effect by inhibiting cell activation (26). In addition to the PD-1/PD-L1 pathway, CTLA-4 is an important immune checkpoint molecule that acts mainly during the initial activation of T cells (27). CTLA-4 inhibits the activity of T cells by preventing co-stimulatory signaling through binding to B7 molecules on the surface of antigen-presenting cells (28). In gastric cancer, tumor cells further enhance T-cell inhibition by upregulating PD-L1 and B7 expression. CTLA-4 acts mainly during initial T-cell activation, whereas PD-1 exerts a sustained inhibitory effect under chronic antigen exposure (29).

#### 1.1.2 Metabolic reprogramming

Metabolic changes in the tumor microenvironment significantly  affect T cell function (30). Gastric cancer cells and immunosuppressive cells (e.g. myeloid-derived suppressor cells, MDSCs) preferentially seize nutrients such as glucose and glutamine through metabolic reprogramming, weakening the metabolic activity of T cells and thus inhibiting their effector function (31). Tumor cells are predominantly aerobic glycolysis, which rapidly consumes glucose and generates large amounts of lactic acid, acidifying the microenvironment, thus further inhibiting T cell proliferation and effector function (32). In addition, lipid accumulation and fatty acid oxidation caused by metabolic reprogramming were enhanced, which promoted the impaired mitochondrial function and accelerated depletion of T cells. Notably, metabolic wastes such as lactate in the tumor microenvironment not only inhibit T cells, but also promote polarization of M2-type tumor-associated macrophages (TAMs) by inducing the expression of HIF-1α, which in turn drives tumor progression (33).

#### 1.1.3 Chronic antigenic stimulation

In gastric cancer, tumor cells continuously express tumor-associated antigens, resulting in a long-term activation state of T cells (34). However, sustained antigenic stimulation not only causes T cells to gradually lose their effector function, but also further deepens the depletion state of T cells by upregulating inhibitory receptors such as PD-1, TIM-3, and LAG-3 (35). It has been pointed out that chronic antigen exposure accompanied by metabolic reprogramming promotes mitochondrial dysfunction, leading to excessive accumulation of reactive oxygen species (ROS) and exacerbating T cell depletion. Therefore, blocking these inhibitory signals or modulating metabolic pathways is expected to restore T cell activity and improve immunotherapy outcomes (36).

### 1.2 T-cell heterogeneity

In the microenvironment of gastric cancer, T cell heterogeneity is mainly reflected in the functional differentiation of subpopulations such as CD8+ effector T cells, memory T cells and regulatory T cells (Treg cells).CD8+ effector T cells (CTLs) are responsible for the direct killing of tumor cells, but they usually show depletion characteristics in gastric cancer patients, which manifests itself in the form of the high expression of inhibitory receptors, such as PD-1, LAG-3, etc., and in this state the CTLs lose their effector function and gradually reduce the secretion of perforin and granzyme, thus failing to effectively kill tumor cells (36). Memory T cells, such as central memory (Tcm) and effector memory (Tem) cells, can be rapidly activated upon re-encountering tumor antigens, but their function is often limited by metabolic defects and inhibitory factors (e.g., TGF-β, IL-10) in the gastric cancer microenvironment, resulting in reduced cell proliferation capacity and secretion of key effector molecules such as IFN-γ. In addition, the number of Treg cells was significantly elevated in gastric cancer tissues, and they suppressed effector T cells by secreting inhibitory factors such as TGF-β and IL-10, and competed with antigen-presenting cells for nutrients, thus weakening anti-tumor immunity (37). In conclusion, the heterogeneity among T cell subsets and their different depletion mechanisms in the microenvironment further reveal the complexity of immune escape in gastric cancer, providing multiple targets and optimized strategies for immunotherapy (38).

## 2 Biomarkers and clinical prediction

As the immune microenvironment of gastric cancer has been studied intensively, a variety of emerging biomarkers have shown significant potential in predicting immunotherapy response and assessing patient prognosis (39).

### 2.1 Emerging biomarkers of T cell dysfunction

CD39/CD73 are key enzymes in adenosine metabolism and are usually highly expressed in depleted T cells. Through the adenosine signaling pathway, CD39 and CD73 contribute to the accumulation of immunosuppressive adenosine in the tumor microenvironment, thereby suppressing the function of effector T cells (40). In gastric cancer patients, high levels of CD39/CD73 expression are closely associated with reduced T cell activity, allowing tumor cells to evade recognition by the immune system. Therapies that block the adenosine pathway, such as A2aR inhibitors, have been shown to restore T-cell function and enhance anti-tumor responses, and are one of the important current research directions in gastric cancer immunotherapy (41).

TIGIT, a newly discovered inhibitory receptor, significantly inhibits the glycolytic activity of CD8+ T cells by binding to its ligand CD155, which in turn reduces the secretion of anti-tumor factors such as IFN-γ (42). Preclinical studies have shown that blocking the TIGIT/CD155 pathway not only restores the metabolic activity of T cells, but also significantly enhances anti-tumor immune responses and improves patient survival. Therefore, the TIGIT/CD155 axis is considered a potential target in gastric cancer immunotherapy with important clinical translational prospects (43).

### 2.2 Biomarkers for predicting response to therapy

Along with the widespread use of immunotherapy in gastric cancer treatment, biomarkers that predict response to therapy are particularly important to help optimize treatment strategies and improve efficacy.

Tumor Mutational Burden (TMB) is considered an important marker of response to immunotherapy (44). A higher TMB usually means more tumor neoantigen generation, enhancing the chances of recognition and attack by the immune system (45). In gastric cancer patients, it has been found that those with higher TMB typically show more significant efficacy to PD-1/PD-L1 inhibitor therapies, as these neoantigens stimulate a stronger immune response (46).

Microsatellite instability (MSI) is another widely studied biomarker in immunotherapy (47). Due to defective DNA mismatch repair, MSI-H (high microsatellite instability) tumors typically have higher mutational loads and neoantigen generation rates, and thus show good response to immune checkpoint inhibitors in gastric cancer.MSI assays have become a commonly used method in the clinic to screen patients for suitability for immunotherapy (48).

In addition, factors such as the number of tumor-infiltrating lymphocytes (TILs) and specific metabolic markers (e.g., CXCL9, IDO, and LDH) have shown significant value in predicting the efficacy of immunotherapy (49). A high density of CD8+ TILs is usually associated with a better clinical prognosis, and high expression of markers such as CXCL9 and IDO predicts a stronger anti-tumor immune response (50). Personalized therapeutic regimens based on these markers can significantly improve the outcome of gastric cancer patients and promote the development of personalized immunotherapy.

## 3 Treatment opportunities and prospects

Immune checkpoint inhibitors (ICIs) and T-cell augmentation therapies have shown unprecedented promise in the treatment of gastric cancer (51). Through immune system modulation, the survival and treatment outcome of gastric cancer patients have been significantly improved (52).

### 3.1 Immune checkpoint inhibitors

In immunotherapy for gastric cancer, PD-1/PD-L1 inhibitors and CTLA-4 inhibitors have been widely used as the two main classes of ICIs (53). Pembrolizumab and Nivolumab are the most common PD-1 inhibitors, which restore the anti-tumor activity of T-cells by blocking the binding of the PD-1 receptor to the PD-L1 ligand (54). For example, the CheckMate 649 trial showed that the combination of Nivolumab and chemotherapy significantly prolonged the overall survival (OS) of gastric cancer patients compared to chemotherapy alone, with a particularly significant survival benefit in patients with high PD-L1 expression (55). In contrast, CTLA-4 inhibitors, such as Ipilimumab, boosted T-cell activity by competitively inhibiting the binding of CTLA-4 to B7 molecules (56, 57). However, due to the low response of gastric cancer to CTLA-4 inhibitors, clinical studies have mostly focused on their use in combination with other therapies to optimize treatment outcomes (58, 59).

In recent years, combination strategies of ICIs with other therapeutic agents have been explored and have shown initial success. For patients with locally advanced gastric cancer, a combination neoadjuvant therapy trial including Karelizumab, Apatinib and chemotherapy showed that patients achieved a complete pathological response rate of 15.8% after surgery, while demonstrating remarkable safety and tolerability. This combination therapy strategy not only acts directly on tumor cells, but also enhances the efficacy of ICIs by inhibiting tumor angiogenesis and elevating the degree of immune cell infiltration in the tumor microenvironment (60).

### 3.2 T-cell enhancement therapy

In addition to ICIs, T-cell augmentation therapies such as chimeric antigen receptor T-cell (CAR-T) therapy and T-cell receptor (TCR-T) therapy have shown great potential in gastric cancer immunotherapy (61).

#### 3.2.1 CAR-T cell therapy

CAR-T therapy targets and kills tumor cells by genetically engineering patient T cells to express receptors for specific tumor antigens (62). For gastric cancer, CLDN18.2-specific CAR-T cells have demonstrated potential efficacy in a phase I clinical trial, with disease control in approximately 48.6% of patients (63). The therapy showed a high overall response rate, especially for gastric cancer patients expressing CLDN18.2, with a 6-month survival rate of 81.2% (63). In addition, ICAM-1-targeted CAR-T cells showed significant anti-tumor activity in gastric cancer models (64). The study showed that by combining with chemotherapy or IL-12, the therapy exhibited significant anti-tumor effects in abdominal metastatic gastric cancer.

#### 3.2.2 TCR-T cell therapy

Unlike CAR-T therapies, TCR-T therapies target more diverse antigens (e.g., tumor-specific mutant antigens) by enhancing the recognition of tumor antigens by T cells (65). In gastric cancer treatment, TCR-T therapy research has focused on targeting antigens such as NY-ESO-1 and MAGE-A4 (66). Although TCR-T therapies have shown some anti-tumor activity in early trials, challenges remain in how to effectively respond to immunosuppression in the tumor microenvironment, especially in the management of off-target effects (67).

## 4 Combination therapy strategies

**Figure 1:**
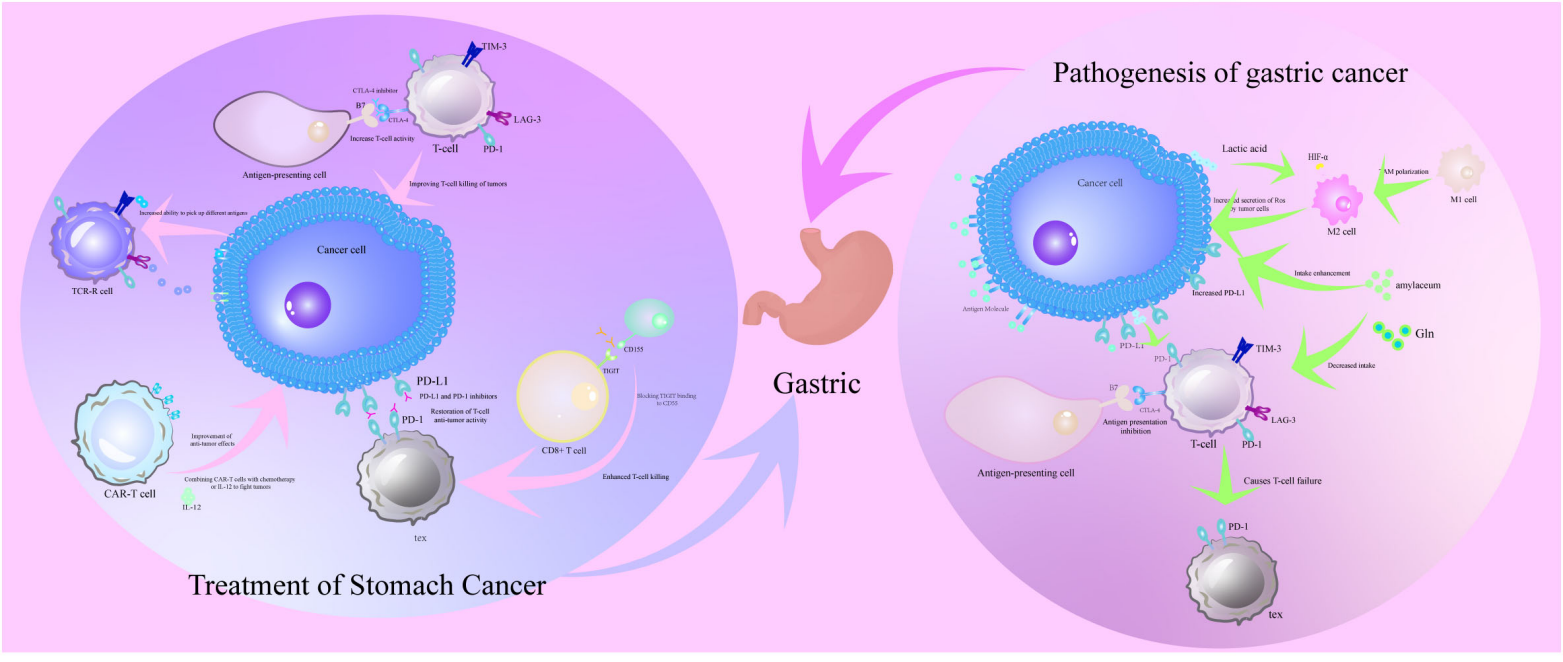
Pathogenesis of T cell dysfunction in the immune microenvironment of gastric cancer and its corresponding therapeutic strategies. The pathogenesis of T cell dysfunction in the immune microenvironment of gastric cancer is the evasion of immune surveillance by gastric cancer cells through metabolic reprogramming of lactate accumulation and HIF-1α activation, recruitment of immunosuppressive cells MDSCs and Tregs, and upregulation of immune checkpoint molecules PD-L1 and TIM-3. Therapeutic strategies for T-cell dysfunction in the immune microenvironment of gastric cancer are immune checkpoint inhibitors PD-1/PD-L1 and CTLA-4 inhibitors restoring T-cell antitumor activity by blocking inhibitory signals, emphasizing the potential of the combined therapeutic strategy of immune checkpoint inhibitors in conjunction with chemotherapy or radiotherapy in improving therapeutic efficacy.

Combination therapy is becoming increasingly important in the treatment of gastric cancer, as monotherapies struggle to overcome the complexity of the tumor microenvironment and multiple drug resistance (68). By combining different therapies, studies have shown that the efficacy of ICIs can be significantly enhanced when combined with chemotherapy, radiotherapy or targeted therapy (69). Chemotherapy and radiotherapy not only kill tumor cells directly, but also enhance the immune system’s ability to recognize and attack tumors by inducing “immunogenic cell death” and releasing tumor antigens (70). These treatments can also alter the tumor microenvironment, reducing the number of immunosuppressive cells such as Treg cells while increasing the infiltration of effector T cells to further enhance the efficacy of ICIs (71). In gastric cancer clinical trials, PD-1 inhibitors combined with chemotherapy or radiotherapy showed significant synergistic effects, significantly prolonging patients’ overall survival (OS) and progression-free survival (PFS) (72). In addition, targeted therapies such as HER2 and VEGFR inhibitors reduce tumor resistance by blocking tumor-specific signaling pathways; studies have shown that the combination of HER2 inhibitors with PD-1/PD-L1 inhibitors significantly activates anti-tumor immune responses and improves prognosis (73). Looking forward, combination therapy will be more based on individual tumor characteristics and microenvironmental status, combined with TMB, MSI and other markers to design personalized programs. In addition, the combination of new immunotherapies such as bispecific antibodies and tumor vaccines with ICIs is also being explored, which is expected to bring more therapeutic options for gastric cancer patients (74) (**Figure 1**).

## 5 Discussion

T cell dysfunction in the immune response to gastric cancer reveals a complex mechanism by which tumors evade immune surveillance (75). In this paper, we systematically review the multiple effects of the gastric cancer microenvironment on T-cell function, focusing on T-cell exhaustion and its resulting immunosuppression. The critical roles of inhibitory pathways such as PD-1 and CTLA-4 in T-cell exhaustion have been intensively investigated, while metabolic reprogramming and chronic antigenic stimulation further exacerbate the loss of T-cell effector function (76). A variety of emerging biomarkers, such as CD39, CD73, and TIGIT, have demonstrated important applications in predicting the response of gastric cancer patients to immunotherapy, providing a basis for individualized stratification and optimization of therapeutic strategies (77).

Although the use of immune checkpoint inhibitors in the treatment of gastric cancer has achieved some success, monotherapy still faces challenges in dealing with the complex tumor microenvironment (78). Combination strategies combining chemotherapy, radiotherapy and targeted therapies have demonstrated significant synergistic effects in the clinic, and future studies should focus on the optimization of these combination regimens and their efficacy differences in different patient populations (79). In addition, although T-cell enhancement therapies such as CAR-T and TCR-T have been successful in hematological tumors, their application in gastric cancer faces challenges such as the lack of tumor-specific antigens and microenvironmental inhibition (80). Personalized, multi-target combination therapy strategies for gastric cancer patients should be the future research direction (81).

In the future, immunotherapy for gastric cancer will further develop towards diversification and precision (82). Through the discovery of novel biomarkers and optimized combinations of treatment strategies, breakthroughs in overcoming drug resistance and improving efficacy are expected. Multidisciplinary collaboration will be key to advancing these studies, ultimately providing patients with a wider range of therapeutic options and longer-term survival benefits. These findings will not only dramatically improve gastric cancer treatment options, but also provide a valuable reference for immunotherapy of other solid tumors.
